# Supplementary material for: Genetic and Physiological Characterization of Soybean-Nodule-Derived Isolates from Bangladeshi Soils Revealed Diverse Array of Bacteria with Potential Bradyrhizobia for Biofertilizers
Source: Microorganisms. 2022 Nov 17;10(11):2282. doi: 10.3390/microorganisms10112282 (PMC9698105; doi:10.3390/microorganisms10112282)
Supplement: Supplementary file 1 [file microorganisms-10-02282-s001.zip › microorganisms-1965717-supplementary.pdf]

Table S1. Information of primers used in this study.

Name	Primer sequences 5'-3'	Reference
1-F	AGTTTGATCCTGGCTC	[23]
3-R	AAGGAGGTGATCCAGCC	
ITS 450	TGYSCACACCGCCCGT	[24]
ITS 1440	CACGTGTYCCGCCBTACT	
F11	CGGAATCAGGCTAAAGCCCTGCACAA	[25]
R11	GGTTCGCGAGGCCCTGCTGCACAT	
Nif H-I	AGCATGTCYTCSAGYTCNTCCA	[26]
Nif H-F	TACGGNAARGGSGGNATCGGCAA	
gyrB343F	TTCGACCAGAAYTCCTAYAAGG	[27]
gyrB1043R	AGCTTGTCCTTSGTCTGCG	
atpD-20F	AGCTTCTTGCCCTTCTCGAC	This study
atpD-1174R	CCGCATCATCAACGTCATC	This study
glnII-9R	ACGGACGAGATCGTCTTCAG	This study
glnII-12F	YAAGCTCGAGTACATYTGGCT	[28]

Table S2 Basic information of soybean varieties used in this study.

	Glycine max cv. Enrei	*Glycine max* cv. Binasoybean-3
**Country**	Japan	Bangladesh
**Release year**	1971	2013
**Weight**	~0.330 gram	~0.085 gram
**Diameter**	~10 mm	~5 mm
**Picture**	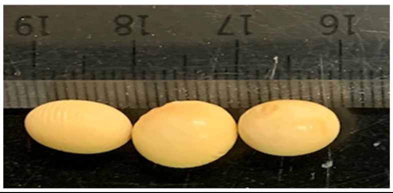	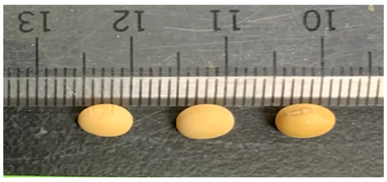

Table S3 List of selected *Bradyrhizobium* isolates for pot experiments and further characterization.

Location	Total Isolated bacteria	16S rRNA gene similarity with known Species	Number of isolates	Number of elected isolates	Selected isolate name	Isolate name (short form)
Bhola	13	*B. liaoningense*	12	1	Bho-P2-B2-S1-51	51
		Pandoraea sputorum	1			
Bogra	1	*B. elkanii*	1	1	Bog-P3-B1-S1-29	29
Dinajpur	13	*B. diazoefficiens*	7	1	Din-P2-M1-M1-25	25
		Methylobacterium tardum	2			
		Pandoraea sputorum	3			
		Stenotrophomonas maltophilia	1			
Lakshmipur	5	*B. elkanii*	3	1	Lax-P1-S1-M1-46	46
		B. liaoningense	1	1	Lax-P1-M1-S1-85	85
		Bacillus pumilus	1			
Mymensingh	6	*B. diazoefficiens*	6	2	Mym-P3-M2-S1-40	40
					Mym-P2-M3-S1-45	45
Natore	12	*B. diazoefficiens*	11	1	Nat-P3-M1-S1-79	79
		Leifsonia shinshuensis	1			
Nilphamary	5	*B. diazoefficiens*	5	1	Nil-P2-B1-M1-36	36
Noakhali	6	*B. diazoefficiens*	6	1	Noa-P1-B1-M1-31	31
Panchagarh	3	*B. liaoningense*	1	1	Pan-P1-B1-S1-69	69
		Bacillus pumilus	2			
Tangail	4	*B. yuanmingense*	2	1	Tan-P1-B2-S1N-84	84
		Pandoraea sputorum	1			
		Stenotrophomonas maltophilia	1			
Thakurgaon	16	*B. diazoefficiens*	10	1	Tha-P2-B1-S1-68	68
		Methylobacterium radiotolerans	4			
		Pandoraea sputorum	2			

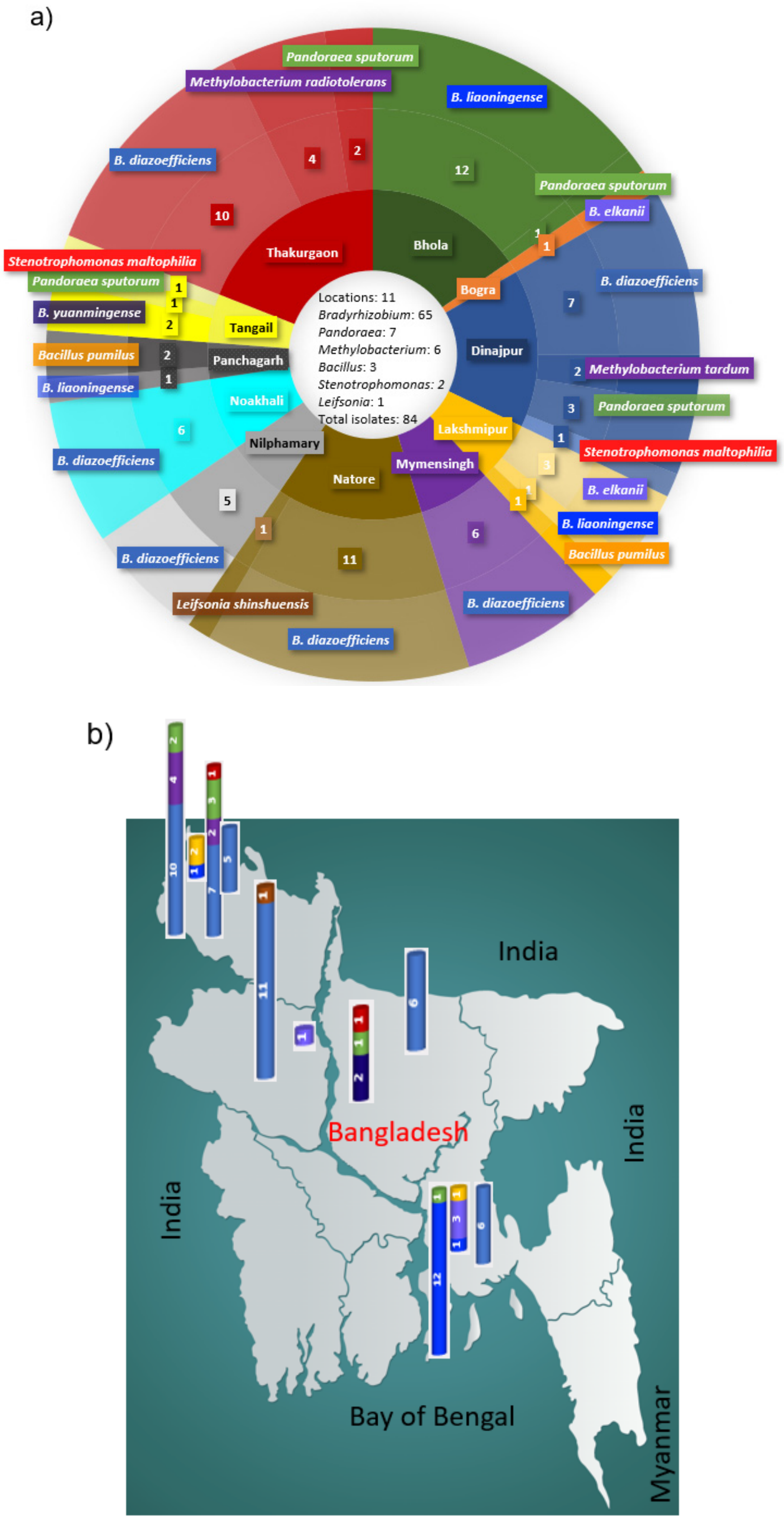

Figure S1. Doughnut graph a) and map of Bangladesh b) showing distribution of different types of bacteria. Colors of the bars in b) are the same as a) showing bacterial species.

Variety	Enrei	BINA	Enrei	BINA	Enrei	BINA	Enrei	BINA	Enrei	BINA	Enrei	BINA	Enrei	BINA
Nodule photo	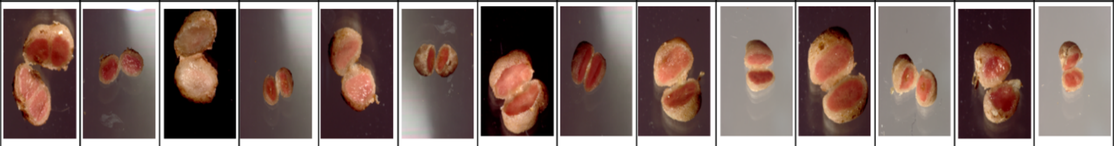													
Nodule number	19±7.810	24±4.359	5±2.646	20±3.606	14±3.606	28±7.550	20.333±1.528	25.333±10.786	18±9.539	20.667±4.933	15.333±4.041	14±2.000	14±2.646	14±2.646
Isolate	USDA110		Noa-P1-B1-M1-31		Tha-P2-B1-S1-68		Din-P2-M1-M1-25		Bho-P2-B2-S1-51		Lax-P1-M1-S1-85		Pan-P1-B1-S1-69	
Genetic similarity	*B. diazoefficiens*		*B. diazoefficiens*		*B. diazoefficiens*		*B. diazoefficiens*		*B. liaoningense*		*B. liaoningense*		*B. liaoningense*	
Nodule photo	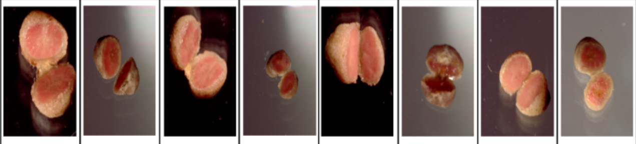								NO Nodule	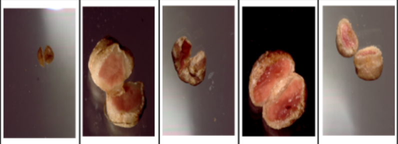				
Nodule number	15.667±2.082	18.667±2.517	10.667±1.528	23.667±5.033	21.333±14.048	22.333±0.577	19.333±2.082	25.333±0.577	13±2.646	18±1.000	21.667±3.055	14.667±3.215	7.667±7.638	23.333±8.505
Isolate	Nil-P2-B1-M1-36		Mym-P3-M2-S1-40		Mym-P2-M3-S1-45		Nat-P3-M1-S1-79		Tan-P1-B2-S1N-84		Lax-P1-S1-M1-46		Bog-P3-B1-S1-29	
Genetic similarity	*B. diazoefficiens*		*B. diazoefficiens*		*B. diazoefficiens*		*B. diazoefficiens*		*B. yuanmingense*		*B. elkanii*		*B. elkanii*	

Figure S2. Pictures of dissected nodules of soybean Enrei variety and Binasoybean-3 variety inoculated with selected isolates and *B. diazoefficiens* USDA110.

a)

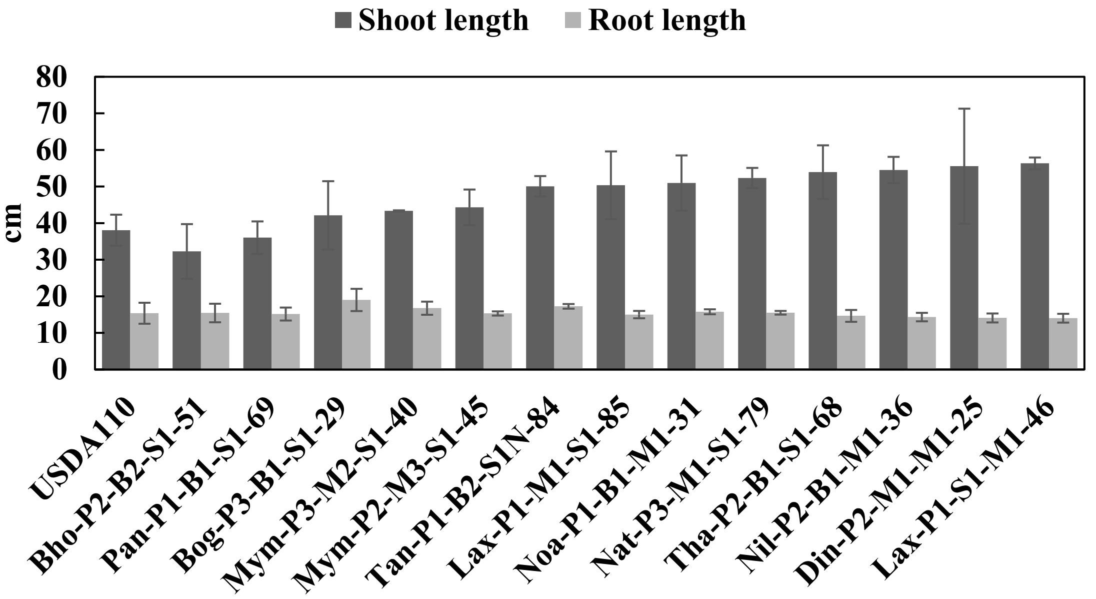

b)

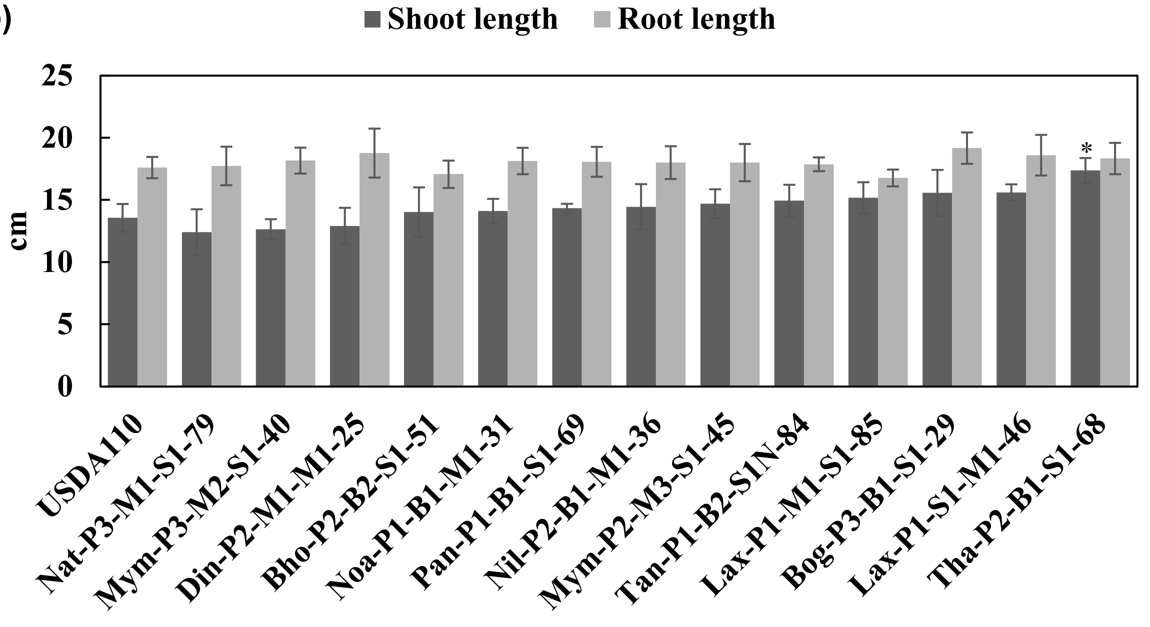

Figure S3. Shoot and root length of soybean Enrei variety a) and Binasoybean-3 variety b) inoculated with selected isolates and *B. diazoefficiens* USDA110. Data are presented in average value with error bar denotes STDEV of three plants each. '*' Denotes significance with "*B. diazoefficiens* USDA110" at 95% confidence level using Dunnett's test.

a)

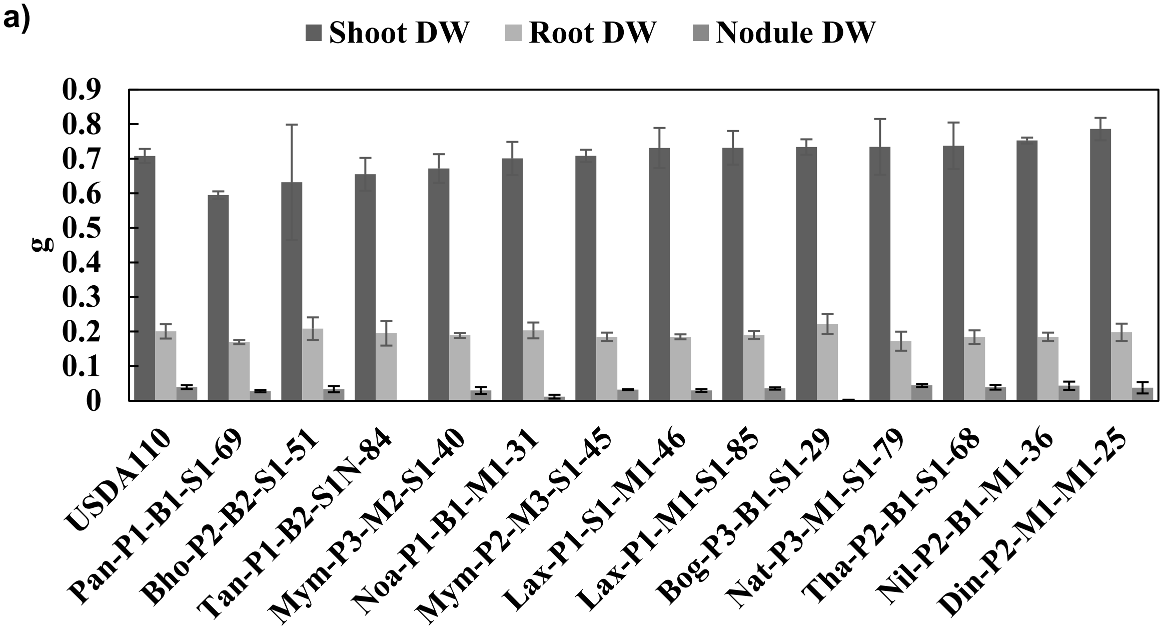

b)

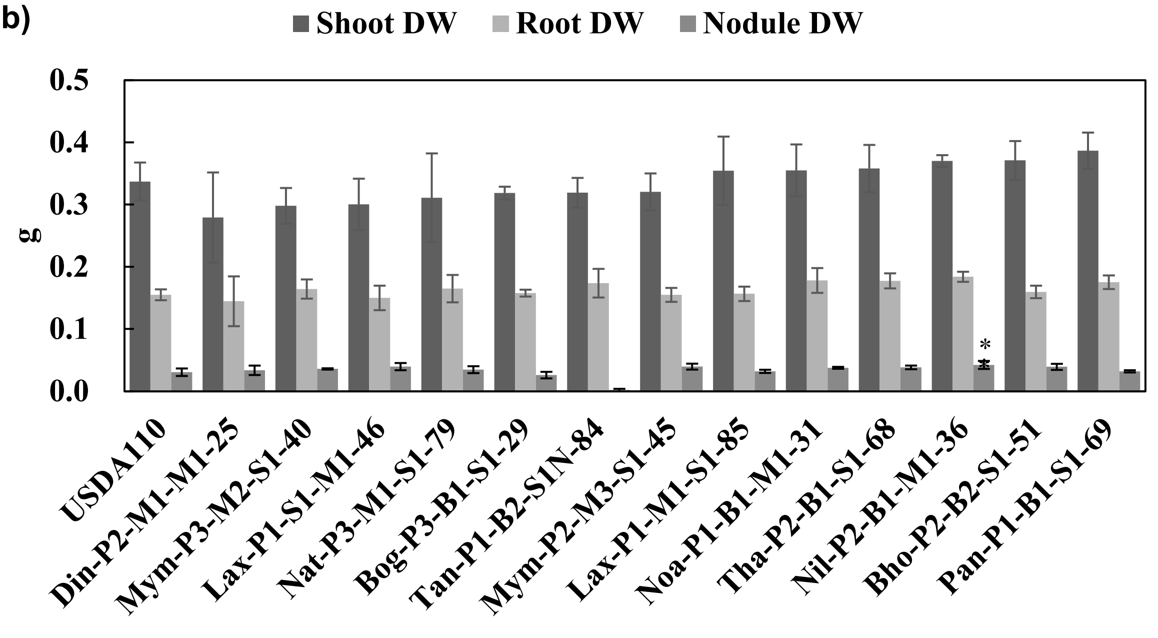

Figure S4. Shoot dry weight (DW), root DW and nodule DW of soybean Enrei variety (a) and Binasoybean-3 variety (b) inoculated with isolates and *B. diazoefficiens* USDA110. Data are presented in average value with error bar denotes STDEV of three plants each. '*' Denotes significance with "*B. diazoefficiens* USDA110" at 95% confidence level using Dunnett's test.
